# Time-Dependent Collagen Fibered Structure in the Early Distraction Callus: Imaging Characterization and Mathematical Modeling

**DOI:** 10.1007/s10439-022-02992-3

**Published:** 2022-06-22

**Authors:** Pablo Blázquez-Carmona, José A. Sanz-Herrera, Juan Mora-Macías, Juan Morgaz, Jaime Domínguez, Esther Reina-Romo

**Affiliations:** 1 Escuela Técnica Superior de Ingeniería, Universidad de Sevilla, Avenida Camino de los Descubrimientos s/n, 41092 Seville, Spain; 2 Escuela Técnica Superior de Ingeniería, Universidad de Huelva, 21007 Huelva, Spain; 3 Hospital Clínico Veterinario, Universidad de Córdoba, Ctra. Nacional IV-A, Km 396, 14014 Córdoba, Spain

**Keywords:** Collagen, Confocal microscopy, Distraction osteogenesis, Orientation, Mathematical modeling, Mineralization

## Abstract

Collagen is a ubiquitous protein present in regenerating bone tissues that experiences multiple biological phenomena during distraction osteogenesis until the deposition of phosphate crystals. This work combines fluorescence techniques and mathematical modeling to shed light on the mechano-structural processes behind the maturation and accommodation-to-mineralization of the callus tissue. Ovine metatarsal bone calluses were analyzed through confocal images at different stages of the early distraction osteogenesis process, quantifying the fiber orientation distribution and mean intensity as fiber density measure. Likewise, a mathematical model based on the experimental data was defined to micromechanically characterize the apparent stiffening of the tissue within the distracted callus. A reorganization of the fibers around the distraction axis and increased fiber density were found as the bone fragments were gradually separated. Given the degree of significance between the mathematical model and previous *in vivo* data, reorganization, densification, and bundle maturation phenomena seem to explain the apparent mechanical maturation observed in the tissue theoretically.

## Introduction

Distraction osteogenesis is a widely-known and mature clinical field that is now being spun out into orthopedic applications, including the treatment of extremities lengthening, bone deformities, or defects.^41,47^ The bases of this procedure are established on a surgical induction of an osteotomy stabilized by an external fixator, a latency period for an initial tissue formation, and a distraction phase when bony fragments are gradually separated.^19^ There are no fixed values for the latency phase duration, rate, and frequency of distraction for all bone models. However, its proper choice is critical to avoid non-unions, bone weakness, or premature consolidation.^17, 19^ A 1 mm/day distraction rate and a latency period between 5 and 7 days are predominant values in long bone experiments.^5, 6, 20, 35, 37^ Once the required length is reached, a consolidation phase begins, including subsequent bone remodeling.

Numerous studies have attempted to unravel the complex biological process behind distraction osteogenesis.^1, 17, 39, 53, 55^ Following an inflammatory response and a hematoma formation after osteotomy, a synthesis of a bone callus matrix occurs in the neighborhood of the osteotomized tissue. The sequential biopsy analysis of Vauhkonen *et al*.^55^ revealed that an organic matrix rapidly fills the distraction gap. Although this matrix is composed of collagenous and non-collagenous proteins, early collagen fibers secreted primarily by osteoblasts become its major component, especially heteropolymers type I.^17, 30, 52, 55^ Thereby, they assume the role of collagen fibers as the primary structural element of the early callus tissue.^26^ This is one of the phenomena activated by the cascade of molecular signals triggered by the mechanical forces involved in distraction.^39^ Not only do the collagen fibers increase in density during the load-induced distraction phase, but they are also gradually aligned in the direction of elongation^17^ and mature structurally. This maturation is understood as the process in which the fibers are crosslinked and packaged to accommodate mineralization.^26^ Thus, a central fibrous interzone is formed in the bone callus. Osteoblasts at this fibrous interzone are also responsible for depositing osteoid on the collagen bundles for further crystallization.^1^ According to Tomoaia and Pasca,^53^ the real mechanism of mineralization is still unknown since collagen cannot induce the formation of the initial amorphous phase of the calcium phosphate.

All this being said, the influence of the distraction loads and the mechanical environment on the callus properties seems undeniable.^27^ This relationship is not particularly new and has been investigated for many years in the field of callus tissue mechanobiology. For instance, the interest in quantifying in a direct way the mechanical properties of the callus led to the appearance of several *ex vivo* studies based on mechanical tests at macro-scale,^40^ or nanoindentation.^2, 28, 34^ Nevertheless, their outcomes are limited by different boundary conditions of the tests and the number of samples and time-points analyzed. Instrumented fixators were also widely used to continuously monitor the distraction forces *in vivo* and assess the correct evolution of the callus ossification process indirectly.^3, 8, 13, 36^ Nonetheless, these studies are generally not able to distinguish between the mechanical behavior of the different tissues involved in the distraction process, including the bone callus, tendons, muscles, or skin. More recently, Blázquez-Carmona *et al*.^6^ decoupled the loss of the surrounding soft tissue viscoelasticity and the mechanical evolution of the elastic fibers and the callus extracellular matrix from experimental data. A stiffening of the callus tissue, understood as an increase in its elastic modulus and a higher resistance to deformation, was also reported during the distraction phase.^6^

According to the biological processes previously described, the main ingredients which are suggested to control this mechanical evolution are the synthesis of new fibers mentioned above, their rearrangement in the direction of traction,^50, 55^ and their maturation. From the author’s point of view, the absence of quantifiable assessments of these parameters is still a major bottleneck in developing more advanced numerical models of distraction than the current ones.^7, 21, 44^ Highly-developed mathematical models could potentially predict the mechanobiological evolution of the bone callus tissue and expand knowledge of the phenomena responsible for tissue stiffening and its accommodation to mineralization during distraction osteogenesis.

Standard histological analyses of soft tissue do not allow proper characterization of the organization of fibrils due to its planar visualization.^30, 48^ Prior studies have also explored a collagenous analysis through scanning electron microscopy. However, its applicability is limited to connective structures made up mostly of collagen (e.g., tendons, joint cartilage, or hydrogels) or isolated fibers.^15, 38, 42^ For more heterogeneous soft tissues, most early approaches succeeded in combining specific dyes, proper staining protocols, image post-processing software, and fluorescence microscopy, including linear polarized light,^59^ confocal,^10^ or multiphoton microscopy.^4, 60^ In the same vein, the objective of the present study is to implement imaging techniques on bone callus tissue for a complete understanding of the geometrical and structural tenets that could affect the evolution of the distraction forces and tissue stiffening. The fiber orientation and density are quantified from image stacks of different interzones of the callus at various time-points of regeneration. These quantitative data are combined in this work with mathematical modeling to further investigate tissue stiffening through the biological phenomena derived from distraction osteogenesis.

## Materials and Methods

### Tissue Preparation

**Figure 1:**
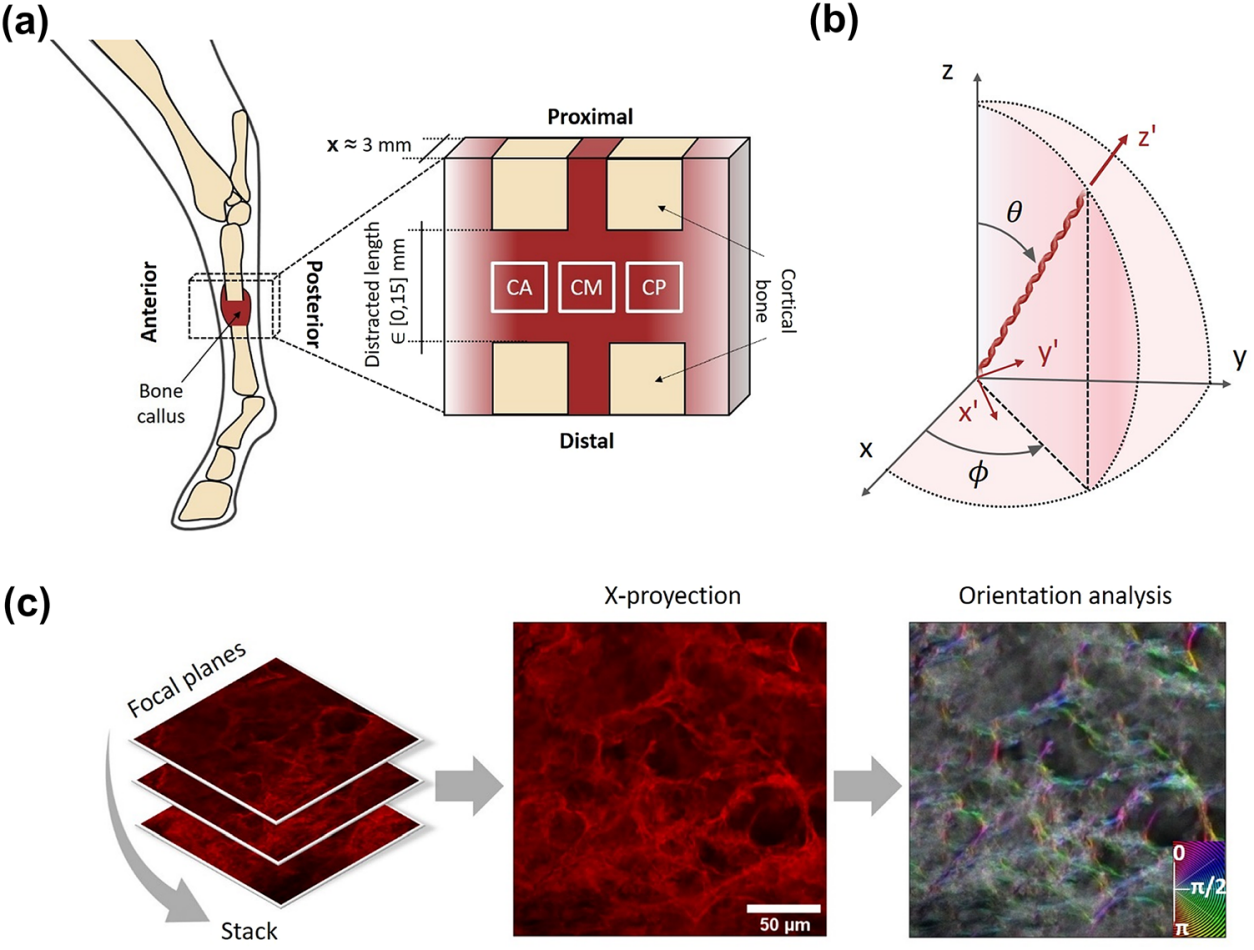
(a) Scheme of the cuts performed to the tissue of each specimen. The three interzones of the bone callus analyzed by fluorescence imaging are also indicated: callus anterior (CA), callus middle (CM), and callus posterior (CP); (b) angle to define a collagen fiber orientation in both global ($xyz$) and local $\left( {x^{\prime}y^{\prime}z^{\prime}} \right)$ configurations: the azimuthal angle $\phi$ and the elevation angle $\theta$; (c) image processing for orientation calculations: acquisition of *x*-stacks, sum-projection, and post-processing through the OrientationJ pluggin^45^.

The samples used in this study come from *in vivo* experiments of distraction osteogenesis in the right metatarsus of six skeletally-mature (3–5 years old) female Merino sheep. Animal use and surgeries were approved by the Animal Ethics of the University of Córdoba (Reference 2021PI/21) following the European (2010/63/UE) and national (RD 1201/2005) regulations. All the specimens followed the same surgical and bone regeneration protocol used in previous studies^5, 6^: osteotomy size of 0.5 mm (blade thickness), a latency period of one week, a distraction rate of 1 mm/day during 15 days, and an Ilizarov-type external fixation.^6^ The fixator was composed of frames attached to the bony fragments using six drilled Schanz-pins and interconnected using extendable bars with a screw-nut mechanism to apply distraction in a controlled manner.^6^ In this study, the animals were slaughtered at different time-points of the bone regeneration process, as specified in Table 1. Given that this study aims to quantify the effects of distraction on collagen fibers before mineral precipitation occurs, all the samples are soft tissue, mainly belonging to the distraction stage. As a reference of a further regeneration stage, a mostly non-mineralized sample of the consolidation phase was also included (day 29 after surgery or day 22 after latency). After sacrifice, treated limbs were frozen at − 80 °C. A longitudinal section, about 3 mm thick, was cut in the sagittal plane of each bony callus (Fig. 1). This plane was selected because no significant mechanical differences were found in the frontal planes of distraction samples from previous studies.^34^ Cuts were performed in the limb fresh out of the freezer using a Femi FM-785XL® band saw (Femi, Castel Guelfo, Bologna, Italy) to ensure the integrity and conservation of the callus soft tissue.

**Table 1:** Experimental information of each bone callus tissue analyzed: days after the latency period, distracted length until the day of sacrifice, and phase of the bone regeneration process.

Animal	Days after latency	Distracted length [mm]	Phase
1	0	0	Distraction
2	3	3	Distraction
3	5	5	Distraction
4	10	10	Distraction
5	15	15	Distraction
6	22	15	Consolidation

As shown in Fig. 1a, three different callus interzones were identified to compare their collagenous structure: callus anterior (CA), callus middle (CM), and callus posterior (CP). Each interzone was manually dissected. Samples were immediately fixed in a buffered 4% paraformaldehyde solution at 4 °C for 3 h and washed three times in cacodylate buffer. Afterward, they were frozen at − 20 °C to cut 100 μm sections using an automatic cryostat Leica RM 2165® (Leica Microsystem, Wetzlar, Germany). A histological stain was used to identify collagen from the rest of the soft tissue. López-Pliego *et al*.^30^ drove a previous histological study in the same ovine metatarsus model subjected to the same biomechanical factors. Intramembranous was reported as the predominant type of ossification in the whole set of calluses at different time-points of the consolidation phase, days 17 to 98 after surgery. The average type I/type II ratio was quantified at 2.48 in these ovine calluses.^30^ Thus, the presence of type II collagen is significantly reduced in an early stage of mineralization and assumed negligible in this work. Consequently, this study focuses on the morphological and quantitative characterization of the type I collagen structures. Picrosirius red (PSR) was selected as an extensively proven histological dye for fluorescence imaging of type I and III of collagen fibers.^4, 56, 59, 60^ All tissue sections were incubated in a one-round PSR staining for 1 h (identical solution and duration) at room temperature and washed twice with distilled water. The non-aqueous dibutyl phthalate polystyrene xylene (DPX) mounting medium was applied before cover slipping. The sample in the consolidation phase (22 days after latency) had a partially ossified CA interzone. Therefore, only CM and CP samples were extracted and analyzed through confocal microscopy.

### Fluorescence Image Acquisition and Data Analysis

Fluorescent microscopy was performed using a laser scanning inverted confocal microscope Zeiss LSM7 DUO® (Carl Zeiss AG, Oberkochen, Germany) with the following objectives: EC Plan-Neofluar 10x/0.30 Plan-Apochromat and Plan-Apochromat 40x/0.95 Corr. The 561 nm DPSS @ 40 mW was fixed as the excitation laser line. The resolutions obtained with these objectives were 1.731 and 6.926 pixels per μm, respectively. The absence of autofluorescence in the tissue was previously verified through unstained sections of the 7-day sample. For each section, *z*-stacks were captured up to the maximum depth at which signal was acquired (Fig. 1c), that varied due to the biological maturation of the tissue.

The fluorescence data analysis was performed using the open-source image processing package Fiji.^49^ The directional analysis of the collagen fibers was carried out on the sum-projection of 40x stacks for a better fibers’ distinction. The OrientationJ plugin was used to evaluate fiber orientation distributions based on the gradient structure tensor in a local neighborhood of each pixel.^45^ A Gaussian-shaped window was fixed to compute the structure tensors by sliding the Gaussian analysis window over the complete projection.^10, 12^ Given that the metatarsal callus is mostly subjected to axial forces due to distraction and limb-loads,^5, 6^ it seems reasonable to find a gradual longitudinal reorientation of the fibers as reported in previous works.^50, 55^ In this way, the preferred orientation was assumed as the direction of distraction owing to the complexity in recognizing the orientation of the specimen during the fixation and mounting processes. Besides, the fluorescence intensity was taken as an indirect measure of the fiber density evolution through the regeneration process. In this case, the maximum projections of the 10 × z-stacks (8-bit images) were used to compare a higher signal volume. Given the difference in depth measured between samples, the same number of intermediate focal planes (4.89 μm z-spacing between planes) was used for this analysis, corresponding to a total depth of 39.13 μm. Indeed, the Mean Gray Value was calculated, which computes the sum of the gray values of all the pixels in the z-projection divided by the number of pixels. In no case the background signal was subtracted from the z-stacks because of the low intensity computed in these image interzones.

### Mathematical Model for Tissue Stiffening

A mathematical model was designed to explain and understand the apparent elastic stiffening of the callus tissue during the distraction phase. Once mentioned their influence in the introduction section, we hypothesized that three key players could govern this problem: the orientation of the fibers around the axial distraction axis (${\eta }_{\theta }$), the collagen and elastic fiber concentration (${\eta }_{d}$), and their bundle maturity (${\eta }_{m}$). In light of the independence of these three structural agents, the axial elastic stiffening was modeled using Eq. 1.

1 $$K\left(t\right)={\eta }_{\theta }\left(t\right)\cdot {\eta }_{d}\left(t\right)\cdot {\eta }_{m}\left(t\right)\cdot {K}_{1}$$

where $t$ is the distraction time after latency ($t$ ∈ [0, 15] days), and ${K}_{1}$ is a constant representing the apparent stiffness of the collagen fibers induced at the end of the latency phase, with the 3D organization corresponding to that time-point (*t* = 0 days) and with a complete maturation (${\eta }_{m}$= 1). The modeling of each of the biologically considered phenomena is detailed below.

The orientation effects on fibered materials are intricate problems involving many factors, including the geometry of the fibers^23, 29^ or the friction contact between the fibers and their surrounding matrix. By integration over the unit sphere, the macroscopic (or apparent) tissue stress in a fibered volume becomes^33^:

2 $${\varvec{\sigma}}_{U} \left( t \right) = \frac{1}{4\pi }\mathop \smallint \limits_{0}^{2\pi } \mathop \smallint \limits_{0}^{\pi } {\varvec{R}}^{T} \left( {\theta ,\phi } \right) \cdot {\varvec{\sigma}} \cdot {\varvec{R}}\left( {\theta ,\phi } \right) \cdot p\left( {\theta ,\phi } \right) \cdot \sin \left( \theta \right)d\theta d\phi$$

where ${\varvec{R}}\left(\theta ,\phi \right)$ is the rotation tensor to the fiber orientation, ${{\varvec{\sigma}}}_{U}$ is the stress tensor at the global configuration (Fig. 1b, $xyz$), ${\varvec{\sigma}}$ is the fiber stress tensor at the local configuration $\left( {x^{\prime}y^{\prime}z^{\prime}} \right)$, $p\left(\theta ,\phi \right)$ is the probability distribution function of the fibers orientation, $\phi (t)$ represents the azimuthal angle of each fiber in the radial plane ($xy$-plane), and $\theta (t)$ is the elevation angle of the fibers to the longitudinal direction of the metatarsus ($z$-axis), at a given time $t$ of analysis. Figure 1b shows both angles defined in Eq. 2. Thereby, ${\varvec{R}}\left(\theta ,\phi \right)$ is defined as^33^:

3 $${\varvec{R}}\left( {\theta ,\phi } \right) = \left( {\begin{array}{*{20}c} {\cos \left( \theta \right) \cos \left( \phi \right)} & { - \sin \left( \phi \right)} & {\sin \left( \theta \right) \cos \left( \phi \right)} \\ {\cos \left( \theta \right) \sin \left( \phi \right)} & {\cos \left( \phi \right)} & {\sin \left( \theta \right) \sin \left( \phi \right)} \\ { - \sin \left( \theta \right)} & 0 & {\cos \left( \theta \right)} \\ \end{array} } \right)$$

Considering this axis configuration, the distraction direction would correspond to the angle *θ* = 0 rad (or $\pi$ rad). Concerning Eqs. 2 and 3, the elasticity in the fiber direction (*z*′) was assumed to control the mechanical properties at the local configuration.^23^ Thus, the normalized fiber orientation coefficient ${\eta }_{\theta }$, which accounts for the mechanical properties in the global $z$-direction, can be defined as:

4 $$\eta_{\theta } = \frac{1}{{4\pi \eta_{\theta ,0} }}\mathop \smallint \limits_{0}^{2\pi } \mathop \smallint \limits_{0}^{\pi } \cos^{2} \left( \theta \right) \cdot p\left( {\theta ,\phi } \right) \cdot \sin \left( \theta \right)d\theta d\phi$$

where ${\eta }_{\theta ,0}$ is the fiber orientation coefficient at the end of the latency phase (*t* = 0 days). The probability distribution function in the unit sphere is normalized as:

5 $$\mathop \smallint \limits_{0}^{2\pi } \mathop \smallint \limits_{0}^{\pi } p\left( {\theta ,\phi } \right) \cdot \sin \left( \theta \right)d\theta d\phi = 1$$

For each distribution obtained from the experimental image analysis, the standard deviation was calculated as the square root of the variance. A linear correlation (as a first approach) of these standard deviations was performed to extrapolate the experimental data to the rest of the distraction days. A homogeneous mechanical behavior was assumed in the whole range of $\phi$ through a uniform distribution. Conversely, anormal probability distribution function for the angular variable $\theta$ between [0, $\pi$] was built for each standard deviation extrapolated from the linear fitting using Matlab® (Mathworks, Natick, MA, US). Note that the probability density function of $\theta$ evolves with time in the process of fiber orientation along the distraction axis. Thus, the integral in Eq. 4 was numerically evaluated (*N* = 10e3) for the evolving temporal $\theta (t)$ probability density functions using Eq. 6:

6 $$\eta_{\theta } \left( t \right) = \frac{1}{{2 \eta_{\theta ,0} }}\mathop \sum \limits_{i = 1}^{N} \cos^{2} \left( {\theta_{i} } \right) \cdot p\left( {\theta_{i} } \right) \cdot w_{i}$$

where ${w}_{i}$ is the numerical weight, $\pi /N$. It should be remarked that ${\eta }_{\theta }\cdot {\eta }_{\theta ,0}$ has the value of 1/12$\pi$ in a random fiber arrangement and 1/4$\pi$ in a fully aligned fiber configuration in the direction of distraction. In line with Kang and Kim,^23^ this fact implies a mechanical contribution three times greater in the case of an alignment between the traction direction and the fibers compared to a random scenario.

On the other hand, the mechanics of bio- or natural-based materials with cellular lattice structures (e.g., collagen-based scaffolds, open-cell foam, balsa wood, or trabecular bone) is generally well documented. In the literature, they are modeled based on the dimensional analysis of deformation mechanisms in their organized porous structures.^14^ Specifically, assuming linear mechanics, the elastic modulus was found to depend on the solid properties and the square of the relative density. In this way, the impact of the collagen density on the callus stiffness through the distraction phase (${\eta }_{d}$) was calculated from the daily fiber density $\rho (t)$ indirectly estimated from a temporal correlation of the mean intensity data (linear fitting as a first approach), and the initial density measured immediately after the latency phase (${\rho }_{o}$):

7 $$\eta_{d} \left( t \right) = C_{1} \cdot \left( {\frac{\rho \left( t \right)}{{\rho_{o} }}} \right)^{2}$$

being the constant ${C}_{1}\sim 15$.^14, 54, 58^

The naïve and induced fibers undergo maturation pathways to consistently increase the stability of the extracellular matrix and acclimate to the mineralization process. This complex process involves wide-ranging mechanisms of collagen crosslinking, packaging, and the cleavage of C- and N-terminal propeptides from the collagen molecules.^25^ Komarova *et al*.^26^ described the composite mineralization problem through ordinary differential equations, including the collagen maturation, the effects of inhibitors, or the mineral nucleation and growth. In particular, the overall maturation effect was modeled with a characteristic constant rate of ${K}_{2}$ = 0.1 day^−1^ for different clinical scenarios, including bone deformities and fractures. This constant was estimated from the long time required for the assembly of the collagen bundles.^9, 26^ Assuming that all fibers are initially naïve at the induction time, the collagen maturation is expressed by the following differential equation:

8 $$\frac{{dm_{c} \left( {t + t^{\prime}} \right)}}{dt} = K_{2} \cdot n_{c} \left( {t + t^{\prime}} \right)$$

where ${n}_{c}$ and ${m}_{c}$ are the normalized concentration of naïve and mature collagen, respectively, and *t*′ is the accommodation-to-mineralization time experienced by the collagen fibers quantified before the distraction phase (during the latency period, *t*′ ∈ [0,7] days). Given the foreseeable continuous induction of collagen due to the mechanical stimulus from distraction, the global maturation coefficient of the bone callus (${\eta }_{m}$) was calculated considering the maturation lag between the fibers from latency. An interpretation of accumulative law, similar to Miner’s fatigue damage accumulation rule, was used^32^:

9 $$\eta_{m} \left( t \right) = \frac{{\mathop \sum \nolimits_{{i = t^{\prime}}}^{t} \rho \left( i \right) \cdot m_{c} \left( i \right)}}{{\mathop \sum \nolimits_{{i = t^{\prime}}}^{t} \rho \left( i \right)}}$$

So then, this mathematical model depends fundamentally on three fitting parameters: ${C}_{1}$, *t*′, and ${K}_{1}$. They were adjusted using the apparent *in vivo* elastic stiffness measured during distraction in previous studies^5, 6, 35^ using Matlab®. In the cited essays, the sheep were subjected to the complete phase of distraction using instrumented fixator bars with load cells Burster® 8431-6001 (Burster, Gernsbach, Germany) in order to quantify the reaction force of hard and soft tissues to distraction. The operated limb was raised to avoid altering the distraction force with internal metatarsal loads. Distraction forces were monitored for 20 minutes after applying the 1 mm bony fragment separation. Blázquez-Carmona *et al*.^6^ also applied the generalized Maxwell rheological model to the raw data so as to discriminate the mechanical behavior of the major components: collagen fibers, extracellular matrix, and surrounding soft tissues (e.g., tendons, muscles, or skin). Hence, the evolution of static callus response after relaxation was associated with the collagen fibers stiffening. These collagen fibers’ stiffening was the basis for the adjustment of the free parameters of the predictive model presented above. More detailed information can be found at Blázquez-Carmona *et al*.^5, 6^ Bound constraints were imposed on fitting the parameter *t*′ in the specified latency time range (0–7 days). Coefficients of determination (*R*^2^ and *p*-value) were calculated to evaluate the significance of every correlation and fitting performed.

## Results

**Figure 2:**
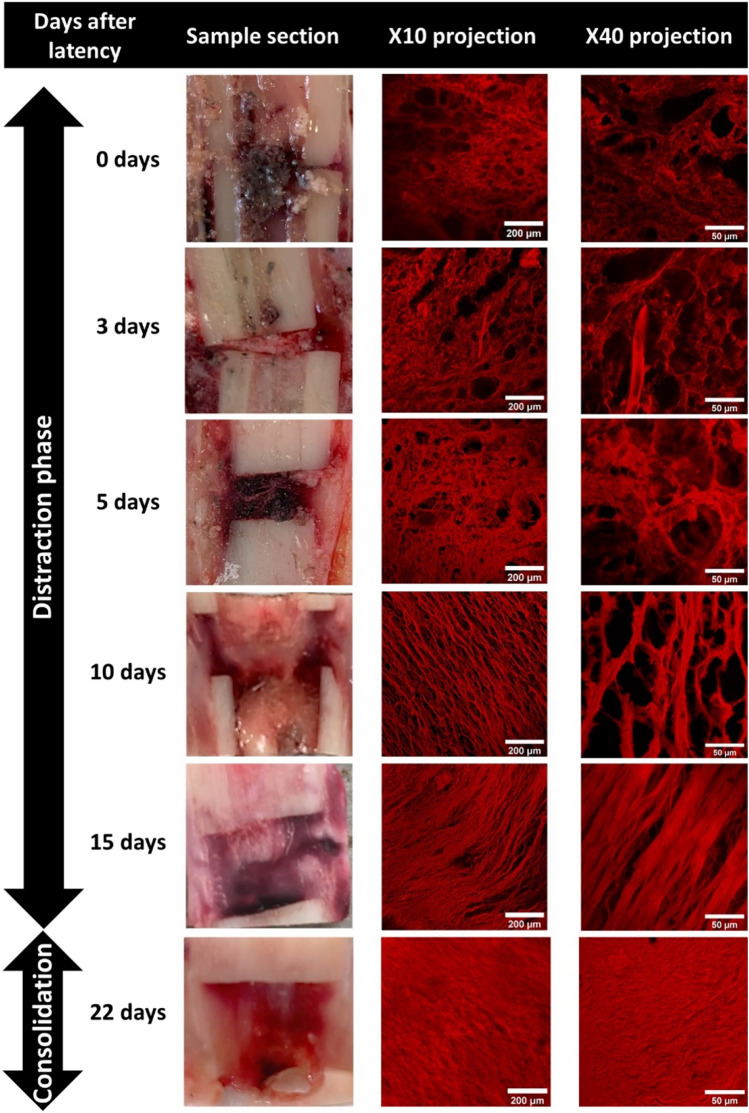
Sum projection of the fluorescence images taken with both × 10 and × 40 objectives and bone callus slices before dissection at different time-points after latency.

Figure 2 shows the temporal evolution of the collagen structure through sum projections of representative stacks taken with both × 10 and × 40 objectives from one callus interzone, and an image of the treated bone slice cut prior to tissue extraction. Qualitatively, the fibers seem to orient in a preferred direction and increase density as cortical bony fragments separate. During consolidation, a broad fluorescence signal can be seen throughout the complete projection.

**Figure 3:**
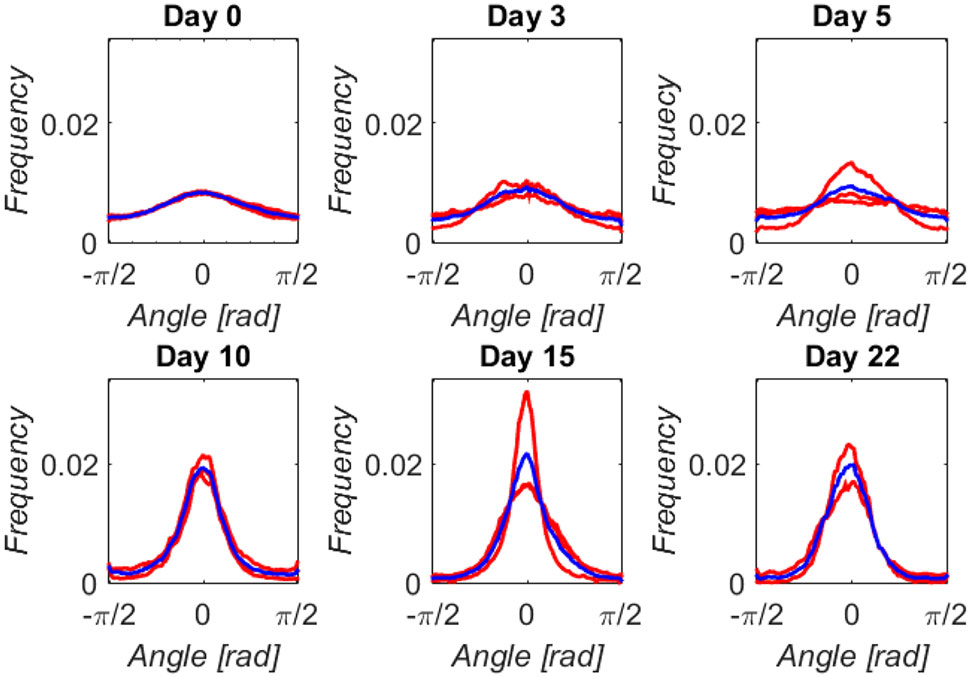
Orientation distribution of collagen fibers at the different time-points analyzed after latency: distribution per callus interzones (red curves) and average between all interzones (blue curve). Distraction axis is *θ* = 0 rad, plotted in the center (− $\pi /2$ to $\pi /2$) for a clearer view on the width of the distribution.

**Figure 4:**
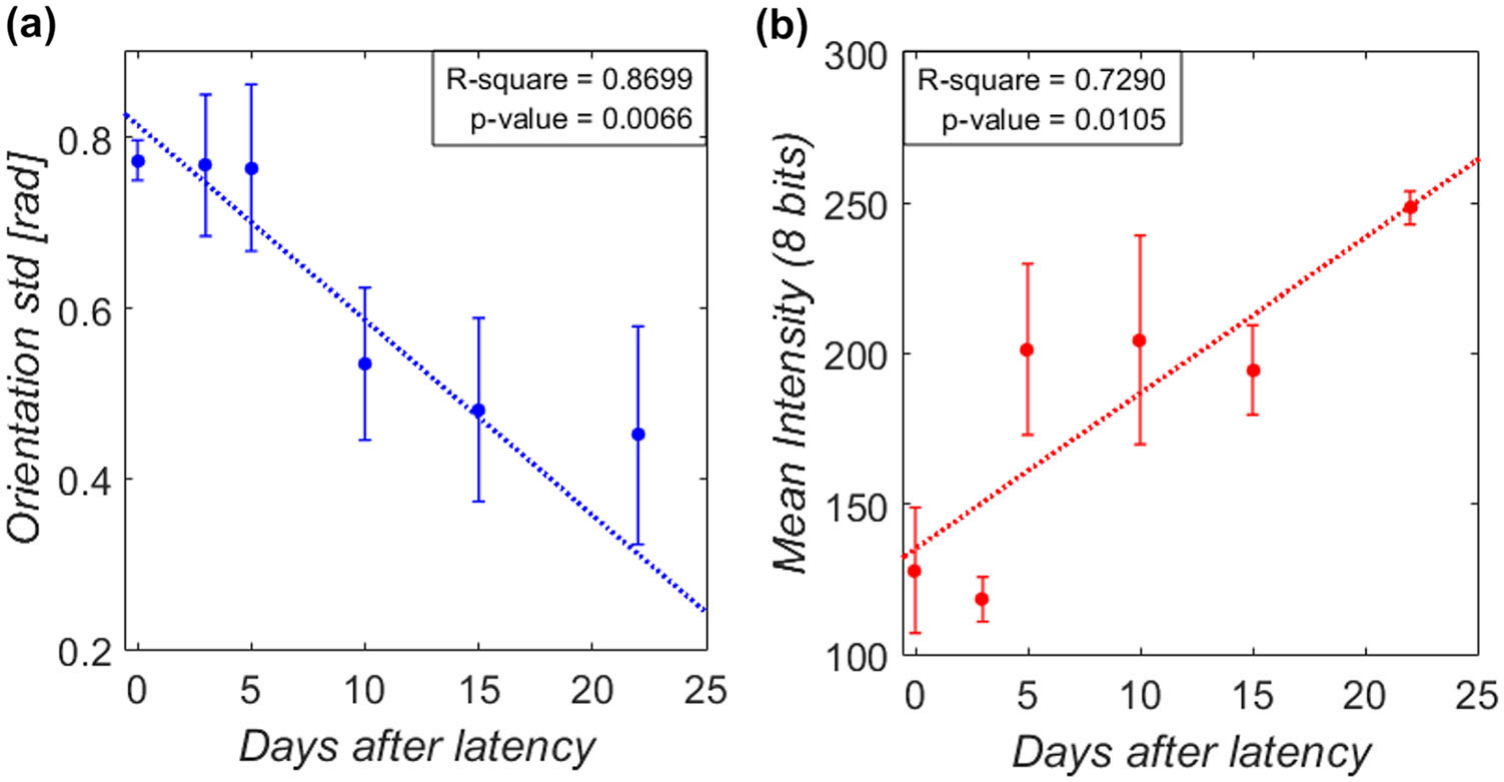
Time evolution of the parameters measured by confocal microscopy (mean and standard deviation per interzones) in the bone callus tissue through the projections of the z-stack fluorescence images: (a) standard deviation of the orientation distribution; (b) mean intensity.

The temporal evolution of the orientation of the collagen fibers is shown in Fig. 3. The normalized frequency orientation distribution for each callus interzone (red curves) and its average (blue curve) over the complete angular range, from − $\pi /2$ to $\pi /2$ rad, are represented for each time-point. In this angular reference, the axial direction of distraction is assumed to be $0$ rad. The first distraction sample reports a practically identical behavior in the three interzones with a random fiber orientation according to the distributions. Like the fluorescence images, the fiber organization tends to a preferred direction as the bony fragments are distracted. In addition, there are slightly more spatial inter-differences within the same bone callus, especially in the CA interzone of the end-of-distraction sample (day 22). In the consolidation tissue, homogeneity in orientation seems to be recovered with a similar average distribution to the previous one. Figure 4a shows the standard deviation of the distributions over the days after latency. Despite the limited analyzed data due to the limited number of specimens and interzones analyzed, this deviation seems to correlate with the regeneration time significantly, *R*^2^ = 0.8699 and *p*-value < 0.01. Concerning the fiber density, the time evolution of the mean intensity is shown in Fig. 4b (*R*^2^ = 0.7290 and *p*-value < 0.01). This quantity undergoes a strong increase after the first days of distraction. However, stabilization is observed in later stages of distraction prior to new growth during early consolidation.

**Figure 5:**
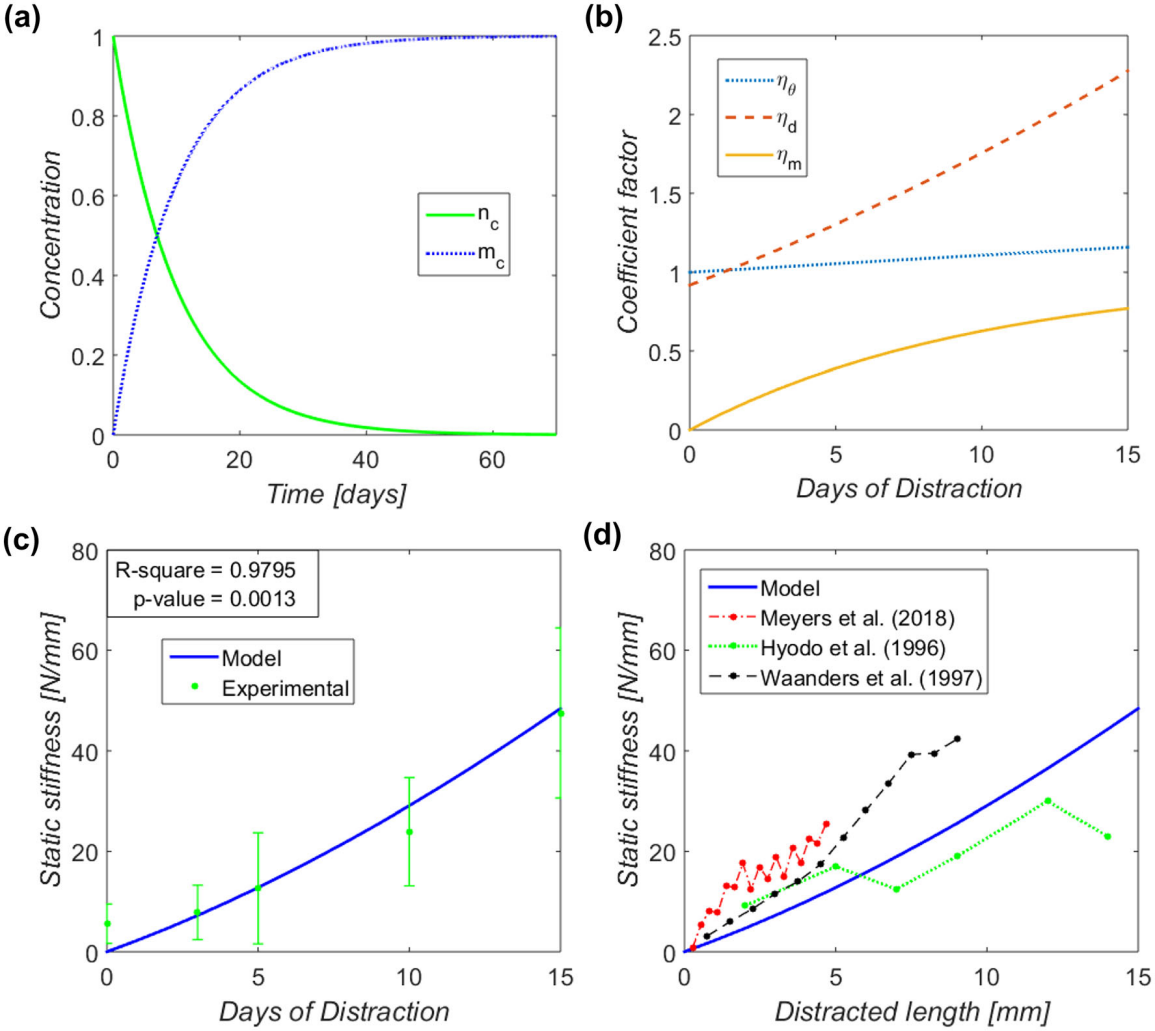
Model outcomes: (a) changes in time in the normalized concentration of naïve (${n}_{c}$, green curve) and mature collagen (${m}_{c}$, blue dotted curve) during a mineralization process^26^; (b) evolution of the multiplicative coefficients of the apparent stiffness of the bone callus through the distraction phase: orientation (${\eta }_{\theta }$, blue dotted curve), density (${\eta }_{d}$, red dashed curve), and maturation (${\eta }_{m}$, yellow curve); (c) comparison between proposed mathematical model of the elastic collagen stiffening during distraction with experimental data from previous *in vivo* works^6, 36^; (d) comparison with other reported data in the literature from Meyers *et al*.^31^, Hyodo *et al*.^18^, and Waanders *et al*.^57^

After fitting of model parameters (which yielded values shown in Table 2), evolution in the normalized concentration of naïve and mature collagen fibers, following the model in Komarova *et al*.,^26^ are represented in Fig. 5a. Despite being a long biological process, half of the fibers are prone to mineralization after a week of maturation (Fig. 5a). Also, changes in the distraction phase in the mathematically modeled stiffening coefficients of the callus tissue are shown in Fig. 5b. Increasing through different behaviors, the orientation, density, and maturation coefficient vary between 1–1.16, 0.91–2.28, and 0–0.77, respectively. In this respect, Fig. 5c compares the evolution of the proposed collagen stiffening mathematical model through the distraction phase and the *in vivo* stiffness of the fibered component of the callus measured by Blázquez-Carmona *et al*.^6^ and Mora-Macías *et al*.^36^ The fitting parameters to achieve this trend are shown in Table 2. Both models and data report a gradual stiffening, reaching around 50 N/mm after 15 days of distraction. Additionally, the coefficient of determination reveals a significant relationship between the experimental data and the defined model: *R*^2^ = 0.9795 and *p*-value < 0.01.

**Table 2:** Fitting parameters in the mathematical model of bone callus tissue stiffening.

Parameter	Description	Value
${C}_{1}$	Constant in Eq. 7	0.92
*t*′	Maturation time before the beginning of distraction	0.15 days
${K}_{1}$	Apparent stiffness of mature fibers after latency	23.87 N/mm

## Discussion

This work provides valuable quantitative data and mathematical models for future *in silico* and *in vitro* research to delve into the role of the mechanics in the mineral precipitation and callus ossification of regeneration processes.

Numerous works had already proved a reorientation of the fibers in the direction of distraction forces through histochemical analysis,^52^ demineralized histology and polarized light microscopy,^22^ or electron micrograph.^43^ Nevertheless, as far as the authors are concerned, no prior study has quantitatively characterized this morphological feature evolution in bone distraction. Fluorescence techniques have been used to evaluate fibers in other soft tissue pathologies, including vocal fold extracellular matrix,^4^ uninjured dermis and scar tissue,^10^ or intervertebral disk degeneration.^60^ The closest research found investigated the distraction of the small intestine.^16^ They reported a more sharply aligner reorientation in the longitudinal direction of the submucosal collagen fibers through the post-operative time. Nevertheless, the changes reported were not as notable as those of the present study, probably due to an initial preferred direction in that tissue. More supporting studies can be found regarding fiber density data. Focusing on the distraction phase, our results show an increase in the proportion of collagen (Fig. 4b). Vauhkonen *et al*.^55^ histologically also reported a growth in the collagen proportion in the total proteins from 53 to 88% during 4 weeks of ovine radius elongation at a lower distraction rate, 0.3–0.5 mm/day. In the same bone model as the present work, López-Pliego *et al*.^30^ described a histological increase from 1.17 to 3.35% in the collagen percentage of the regenerated tissue between the last 5 days of distraction. A similar fiber density growth rate is measured in the current study according to the coefficient ${n}_{c}$ (Fig. 5b), being 2.48 during the complete distraction phase.

The mathematical modeling of the elastic callus stiffening (Fig. 5) also provides interesting insights. The achieved level of fitting significance (*R*^2^ = 0.9795 and *p*-value < 0.01) between the model and the experimental data from Blázquez-Carmona *et al*.^6^ and Mora-Macías *et al*.^35^ shows that, in different degrees, the three modeled coefficients (orientation, density, and maturation) play a fundamental role in the collagenous network mechanical response. Collectively, a similar pattern of results was obtained in other works in the broader literature. As a composite, many existing studies have examined a clear influence of fiber orientation on the mechanics of several biological materials, including cortical bone^46^ or collagen gels.^51^ Kanungo and Gibson^24^ also reported an *in vitro* relationship between density and mechanical properties in collagen–glycosaminoglycan scaffolds. For example, their compressive elastic modulus and strength in a dry state shifted with the density from 32 to 127 kPa and from 5 to 19 kPa, respectively. Regarding the maturation coefficient, Depalle *et al*.^11^ described an *in silico* reinforcement of the elastic modulus, maximum stress, and toughness as the density of immature divalent or mature trivalent crosslinks grows. Otherwise, the fitting values of the parameters specified in Table 2 appear to have consistency when compared with other fittings in the literature. The constant of the density coefficient ${C}_{1}$ approaches 1, as found by Warren and Kraynik^58^ in a tetrakaidecaheral unit cell model or by Vajjhala *et al*.^54^ in three-dimensional Voronoi foams. Before the first distraction test, the maturation time was relatively low, *t*′ = 0.15 days. This suggests that the maturation process of the fibers occurs mainly during the distraction phase, probably because latency biology focuses primarily on hematoma formation surrounded by fibroblasts.^17^ Finally, the initial tissue stiffness (${K}_{1}$ = 23.87 N/mm) is in line with the provided *in vivo* data in the first days of distraction.^6, 36^

Comparing the mathematical model against other experimental data is not an immediate issue due to the wide variety of bone models and distraction protocols applied in the literature.^18, 31, 57^ Figure 5d compares the modeled elastic callus stiffening over the total distracted length with other stiffness data built from studies which reported static distraction force data and the applied distraction rate. Despite the mentioned interdifferences, no significant dissimilarities were observed. Meyers *et al*.^31^ applied distraction on an ovine tibia model using an axial stimulation lateral fixation. Their overall higher mechanical properties could be explained by using a more vascularized bony model and a longer latency time, 10 days vs. 7 days in our study. The canine femur lengthening of Hyodo *et al*.^18^ experienced a slightly lower stiffness by imposing a distraction rate of 1 mm/day. Therefore, applying the same distraction rhythm to a smaller bony model is suggested to harm the structure and maturation of the fibers. Finally, the faster stiffening in the Waanders *et al*.^57^ could be due to assuming a precipitate absence of force relaxation beyond their reported 9-min resting force data. Future studies could fruitfully explore the relationship between the tissue mechanical environment and their structural arrangement further in order to understand their implications on mineralization.

During the consolidation phase, wide-ranging mechanisms controlling bone ossification take place. According to previous microradiology and non-decalcified histology studies,^1^ five zones can be found in the bone callus at this stage: a central fibrous matrix, two peripheral mineralized zones, and two intermediate zones of proliferating cells commonly referred to as ’mineralization front’. In the analyzed consolidation sample, the two ossification centers probably started in the ossified CA interzone. The lack of homogeneity in mineralization concerning the other two callus interzones analyzed by microscopy, CP and CM, is striking. Relative to Fig. 3, no reorganization of fibers was reported with respect to the last distraction sample after the end of distraction forces. However, fluorescence images and intensity data (Fig. 4b) show a remarked increase in the fiber network through self-assembly, probably settling for the impending apatite precipitation.^53, 55^ This continuous collagen synthesis beyond the distraction phase again agrees with the ovine callus histology of López-Pliego *et al*.,^30^ which quantified a 4.01% of type I collagen in the 0.34 mm^2^ analyzed field at day 98 after surgery. Tay *et al*.^52^ also supported these results by reporting cells within the fibrous callus interzone expressing collagen and exhibiting abundant alkaline phosphatase activity prior to differentiation in the maturation phase. In spite of the potential interdifferences between specimens, this phenomenon could explain a less acute preferential orientation in one callus interzone. The lack of a distraction stimulus could trigger a more random organization in the new fibers induced during the consolidation phase.

Although the mathematical modeling of the elastic stiffening of the callus focuses on the distraction phase, Blázquez-Carmona *et al*.^5^ proposed a model to estimate the callus stiffness *in vivo* by monitoring the ground reaction forces and the forces through the external fixator during consolidation. The continuous exponential stiffening reported in that study could be due to the heterogeneous combination of mineralization, collagenous densification, and maturation. Due to the limited number of specimens in the current study, it is unclear whether the formation of the mineralization centers always appears in the CA interzone. This is an interesting open question for future research that could be explored throughout different biomechanical factors: rate of distraction, frequency of distraction, or distraction length.
